# Socioeconomic and ethnic disparities in breast cancer-related lymphedema and quality-of-life after immediate lymphatic reconstruction

**DOI:** 10.1007/s10549-025-07829-w

**Published:** 2025-11-20

**Authors:** Abbas M. Hassan, John P. Hajj, John P. Lewis, Carla S. Fisher, Folasade O. Imeokparia, Kandice K. Ludwig, Rachel M. Danforth, R. Jason VonDerHaar, Ravinder Bamba, Mary E. Lester, Aladdin H. Hassanein

**Affiliations:** 1 Division of Plastic Surgery, Indiana University School of Medicine, Indianapolis, Ind. USA; 2 Division of Breast Surgery, Indiana University School of Medicine, Indianapolis, Ind. USA

**Keywords:** ILR, Lympha, Lymphedema, Breast cancer, ADI, Ethnicity, Socioeconomic disadvantage

## Abstract

**Purpose:**

Breast cancer-related lymphedema (BCRL) disproportionately impacts patients facing socioeconomic challenges. The influence of socioeconomic disparities on preventive procedures such as immediate lymphatic reconstruction (ILR) is unclear. We sought to determine the impact of area deprivation index (ADI) on BCRL incidence and patient-reported outcomes (PROs) following ILR.

**Methods:**

We retrospectively studied consecutive patients who underwent ILR following ALND between 2017 and 2024 across multiple hospitals within a hospital network. Patients were stratified into quartiles based on ADI (Q1 = least deprived, Q4 = most deprived). BCRL prevalence and condition-specific (LYMPH-Q) quality-of-life performance was compared and correlated across quartiles via multivariable regression, including subgroup analysis by ethnicity.

**Results:**

We identified 172 patients with follow-up time of 23.1 ± 15.2 months. Patients residing in the most deprived neighborhoods (ADI Q4) demonstrated significantly higher BCRL rates compared to those from less deprived neighborhoods (Q1-3) (16.3% vs. 3.9%; *p* = 0.006). In multivariable regression, residence in the most deprived neighborhoods remained independently associated with a significantly higher risk of BCRL (OR 5.10, 95% CI 1.30–20.30; *p* = 0.021). Subgroup analysis revealed that Black patients in the highest ADI quartile reported significantly worse LYMPH-Q function scores (median 62.0 vs 100.0; p = 0.020), compared to Black patients residing in less deprived areas. ADI was not significantly associated with surgical complications or unplanned reoperations.

**Conclusions:**

Neighborhood socioeconomic disadvantage significantly increases BCRL risk following ILR and is associated with significantly worse patient-reported functional outcomes among Black patients. Targeted interventions addressing neighborhood-level factors are critical to mitigate these disparities and ensure equitable outcomes.

## INTRODUCTION

Immediate lymphatic reconstruction (ILR) is a microsurgical procedure performed concurrently with axillary lymph node dissection (ALND) to mitigate the risk of breast cancer-related lymphedema (BCRL) [1–4]. This procedure involves anastomosing disrupted afferent lymphatic vessels in the axilla to nearby veins to prevent lymphedema [5–7]. Prior studies have reported significantly lower lymphedema rates in patients undergoing ILR at the time of ALND compared to those without ILR [5–17].

Social determinants of health (SDoH), particularly as captured by the Area Deprivation Index (ADI), play a critical role in influencing surgical outcomes [18–20]. The ADI is a composite measure of neighborhood-level socioeconomic disadvantage, incorporating factors such as income, education, employment, and housing. Previous research has demonstrated that patients residing in more deprived neighborhoods, as indicated by higher ADI scores, often experience higher mortality rates and worse surgical outcomes. These include higher rates of complications, readmissions, and worse patient-reported outcomes mediated by mechanisms including material deprivation, psychosocial stressors, maladaptive health behaviors, and limited access to essential resources [21–27].

Although research has explored the association between SDoH and BCRL, suggesting underdiagnosis in disadvantaged individuals [28], the impact of these factors on long-term BCRL outcomes, particularly after ILR, has not been studied. Effective management of BCRL often requires consistent access to resources such as specialized therapists, compression garments, all significantly influenced by socioeconomic circumstances. Despite understanding SDoH’s impact on BCRL diagnosis, a critical knowledge gap exists regarding its influence on prophylactic interventions like ILR. Determining the influence of socioeconomic factors on BCRL incidence following ILR is crucial to ensure equitable benefits from this technique.

The purpose of this study is to address this knowledge gap by evaluating the long-term association between socioeconomic disadvantage, as measured by ADI, and ILR outcomes across multiple hospitals in a university hospital network. We hypothesized that patients from more deprived neighborhoods will experience higher rates of BCRL and have worse patient-reported quality-of-life.

### Methods

#### Study design and participants

This retrospective cohort study included adult female patients (> 18 years old) who underwent immediate lymphatic reconstruction (ILR) at the time of ALND during breast cancer treatment at six University-affiliated hospitals within our institution between 2017 and 2024. Exclusion criteria comprised patients who had undergone only sentinel lymph node biopsy (SLNB), pre-existing lymphedema, limb swelling from secondary etiologies such as deep venous thrombosis or chronic venous insufficiency, inadequate follow-up data, or incomplete residential address data required for geocoding. The University Institutional Review Board approved this study.

#### Covariables

Patient data collected from electronic medical records included demographic factors (age, ethnicity, body mass index [BMI], tobacco usage, American Society of Anesthesiologists [ASA] classification), cancer-related data (type of cancer, nodal status including number removed and number positive), comorbidities (diabetes mellitus, hypertension, hyperlipidemia, chronic obstructive pulmonary disease), and treatments administered (chemotherapy and radiotherapy, preoperatively or postoperatively, and specific radiation locations: axillary, chest wall/breast, supraclavicular, internal mammary). Surgical data were recorded, including breast surgery type, reconstruction methods and timing, and the number of lymphovenous bypasses performed during ILR. Tobacco use was categorized as active use within eight weeks prior to ALND, while obesity was defined as BMI greater than 30 kg/m^2^.

#### ADI stratification

Residential addresses obtained from patient records were geocoded and linked to corresponding census block groups to assign national Area Deprivation Index (ADI) percentile scores, similar to published methods [27]. Briefly, ADI scores reflect socioeconomic deprivation using 17 indicators such as education, employment, housing, and poverty status [24, 29, 30]. Scores range from 1 to 100, with higher scores indicating greater disadvantage [24, 30]. To facilitate our analysis, patients were categorized into four ADI quartiles, with Q1 indicating the lowest deprivation and Q4 indicating the highest deprivation [22, 24, 29–32]. Comparative analyses were designed to evaluate outcomes between patients in the highest deprivation quartile (Q4) and those in quartiles Q1-3.

#### Study outcomes

The primary endpoint was breast cancer-related lymphedema (BCRL) incidence following ILR, identified by a ≥ 2 cm circumference difference between the operated and non-operated limbs at two adjacent measurement points [33]. Secondary endpoints included postoperative patient-reported outcomes, surgical complications, and unplanned reoperation. Preoperative and postoperative consultations by physical or occupational therapists included baseline limb measurements and continuous postoperative monitoring for signs of lymphedema.

#### ***Surgical technique***

Five microsurgeons performed ILR using standardized protocols described previously [6, 34, 35]. Briefly, lymphatic vessels were identified using intraoperative dyes (isosulfan blue or fluorescein), and lymphovenous anastomoses were created via the sleeve technique [34]. ALND procedures were performed by eight breast surgeons using standard oncologic surgical approaches either via primary mastectomy incisions or separate axillary incisions. Surgical drains were routinely placed postoperatively [36].

#### Patient-reported outcomes

We evaluated patient-reported outcomes using the LYMPH-Q, a validated patient-reported outcome measure (PROM) specifically designed to assess quality-of-life related to upper extremity lymphedema following breast cancer treatment [37]. Four scales from the LYMPH-Q Upper Extremity Module were administered to the study population in March 2025: Symptoms, Function, Psychological, and Appearance. Items for each scale were converted to an overall score ranging from 0 to 100 using the corresponding conversion tables, with a higher score indicating a better outcome (e.g., fewer symptoms, better function). Three electronic reminders were sent in the event of a nonresponse, and no monetary or in-kind incentive was provided to participants.

#### Statistical analysis

Categorical data were summarized using frequencies and percentages and analyzed using chi-squared or Fisher’s exact tests. Continuous data were reported as means (with standard deviations) or medians (with interquartile ranges), depending on the distribution, and analyzed using t tests or Mann–Whitney U tests as appropriate. Logistic regression models (univariate and multivariable) were employed to identify the associations between ADI quartiles and BCRL, with odds ratios (ORs) and 95% confidence intervals (CIs) calculated. Multivariable regression models were optimized using stepwise selection criteria based on the lowest Akaike information criterion (AIC). All statistical tests were two-tailed, with statistical significance set at *p* < 0.05. Data analyses were conducted using SAS Enterprise Guide, version 9.4 (SAS Institute Inc., Cary, NC, USA).

## Results

### Patient demographics and characteristics

We identified 172 consecutive patients during the study period who met the inclusion criteria with mean age 50.9 ± 11.6 years, BMI of 29.5 ± 6.9 kg/m^2^, and follow-up of 23.1 ± 15.2 months. Complete residential address data were available for ADI geocoding in 170 patients (98.8%). Most patients were White (*n* = 134; 77.9%), classified as ASA class 3 (*n* = 95; 55.2%), and diagnosed with infiltrating ductal carcinoma (*n* = 127; 73.8%). Patients were distributed across ADI quartiles as follows: 28.2% in the lowest deprivation quartile (Q1), 23.5% in Q2, 22.9% in Q3, and 25.3% in the highest deprivation quartile (Q4).

### Area deprivation index and patient characteristics

Table 1 summarizes baseline patient characteristics stratified by ADI quartiles. Patients residing in neighborhoods within the highest deprivation quartile (ADI Q4) were significantly more likely to identify as Black (37.2%) compared to lower deprivation quartiles (12.6%; *p* = 0.004). Conversely, White (62.8% vs. 82.7%) and Asian patients were significantly less represented in the highest deprivation quartile. Additionally, chronic obstructive pulmonary disease (COPD) was significantly more common among patients residing in the most deprived areas (9.3%) compared to patients from less deprived quartiles (0.8%; *p* = 0.004). No other significant demographic differences were identified across ADI quartiles.

**Table 1:** Patient demographics according to area deprivation index quartiles

	All pts	Area deprivation index groups		p Value
		Q1-3	Q4	
Patients, n (%)	170	127 (74.7)	43 (25.3)	
Age, y, mean ± SD	50.9 ± 11.6	50.9 ± 11.1	50.7 ± 12.2	0.945
Body mass index, mean ± SD	29.5 ± 6.9	29.4 ± 7.3	30.0 ± 5.7	0.582
Obesity, n (%)	72 (41.8)	53 (41.7)	18 (41.2)	0.988
Ethnicity, n (%)				**0.001**
White	132 (77.7)	105 (82.7)	27 (62.8)	
Black	32 (18.8)	16 (12.6)	16 (37.2)	
Asian	6 (3.5)	6 (4.7)	0 (0.0)	
ASA classification, n (%)				0.218
2	77 (45.3)	61 (48.0)	16 (37.2)	
3	93 (54.7)	66 (52.0)	27 (62.8)	
Length of follow-up, mo, mean ± SD	23.1 ± 15.2	22.6 ± 14.3	24.6 ± 17.9	0.509
Tobacco use, n (%)	17 (10.0)	10 (7.9)	7 (16.3)	0.112
Medical comorbidity, n (%)				
Hypertension	49 (28.8)	35 (27.6)	14 (32.6)	0.532
Diabetes mellitus	25 (14.7)	17 (13.4)	8 (18.6)	0.404
Hyperlipidemia	41 (24.1)	34 (26.8)	7 (16.3)	0.164
Chronic obstructive pulmonary disease	5 (2.9)	1 (0.8)	4 (9.3)	**0.004**
Cancer type, n (%)				0.351
Infiltrating ductal carcinoma	125 (73.5)	97 (76.4)	28 (65.1)	
Infiltrating lobular carcinoma	18 (10.6)	12 (9.5)	6 (14.0)	
Other	27 (15.9)	18 (14.2)	9 (20.9)	

*ASA* American society of anesthesiologists

### Surgical characteristics

Table 2 presents surgical characteristics stratified by ADI quartiles, respectively. The majority of patients (57.7%, *n* = 97) underwent mastectomy and ALND with breast reconstruction. Mastectomy and ALND without reconstruction was performed in 26.2%, while lumpectomy with ALND without reconstruction was done in 16.1%. Tissue expander reconstruction was the most common modality (44.8%, *n* = 77). No significant differences were observed across ADI groups in receiving neoadjuvant chemotherapy, adjuvant chemotherapy, or radiotherapy treatments. Additionally, no significant differences were noted regarding reconstruction modality, timing of reconstruction, lymph nodes removed, or the number of lymphovenous bypasses performed.

**Table 2:** Surgical characteristics according to area deprivation index

Variable	All pts	Area deprivation index groups		*p *Value
		Q1-3	Q4	
Patients, n (%)	170	127 (74.7)	43 (25.3)	
Surgery type, n (%)				0.205
Lumpectomy and ALND	26 (15.7)	17 (13.6)	9 (22.0)	
Mastectomy and ALND	44 (26.5)	31 (24.8)	13 (31.7)	
Mastectomy, ALND and breast reconstruction	96 (57.8)	77 (61.6)	19 (46.3)	
Chemotherapy, n (%)				
Preoperative	91 (54.2)	64 (50.8)	27 (64.3)	0.129
Postoperative	41 (24.4)	31 (24.6)	10 (23.8)	0.917
Postoperative radiotherapy, n (%)	138 (82.6)	105 (84.0)	33 (78.6)	0.422
Radiation location, n (%)				0.152
Axillary and breast/chest wall	40 (28.6)	27 (25.5)	13 (38.2)	
Axillary, breast/chest wall with supraclavicular and/or internal mammary radiation	100 (71.4)	79 (74.5)	21 (61.8)	
No. lymph nodes removed during ALND, mean ± SD	15.0 (10.0–21.0)	15 (10.0–21.0)	18.5 (12.0–22.0)	0.920
No. positive lymph nodes removed during ALND, Median (IQR)	2.0 (0.0–4.0)	2.0 (1.0–4.0)	2.0 (1.0–5.0)	0.359
No. lymphovenous bypasses performed, Median (IQR)	1 (1.0–4.0)	1.0 (1.0–4.0)	1.0 (1.0–4.0)	0.791
Breast reconstruction type, n (%)				0.143
None	74 (43.5)	50 (39.4)	24 (55.8)	
Prosthetic	76 (44.7)	62 (48.8)	14 (32.6)	
Autologous	20 (11.8)	15 (11.8)	5 (11.6)	
Timing of reconstruction, n (%)				0.841
Immediate	13 (13.5)	10 (13.0)	3 (15.8)	
Delayed	17 (17.7)	13 (16.9)	4 (21.1)	
Staged	67 (68.8)	54 (70.1)	12 (63.1)	

### Surgical outcomes

Surgical outcomes stratified by ADI quartiles are presented in Table 3. Patients in the highest deprivation quartile (ADI Q4) demonstrated significantly higher rates of BCRL compared to patients from lower deprivation quartiles (16.3% vs. 3.9%; *p* = 0.006). In univariate analysis, no significant differences were observed in surgical complications (*p *= 0.410, *p* = 0.443), or unplanned reoperation (*p* = 0.559, *p* = 0.893), when stratified by ADI. Multivariable parsimonious logistic regression models assessing the impact of ADI on study outcomes are shown in Table 4. After adjusting for ethnicity, COPD, and number of lymph nodes removed during ALND, residing in the highest deprivation quartile was independently associated with increased odds of developing BCRL (OR 5.10, 95% CI 1.30–20.30; *p* = 0.021). ADI was not significantly associated with surgical complications or unplanned reoperations in multivariable analyses.

**Table 3:** Surgical outcomes according to area deprivation index

Variable	All pts	Area deprivation index groups		*p* Value
		Q1-3	Q4	
Patients, n (%)	170	127 (74.7)	43 (25.3)	
Lymphedema	12 (7.1)	5 (3.9)	7 (16.3)	**0.006**
Time to significant limb swelling, months, Median (IQR)	4.5 (1.0–11.3)	4.0 (0.0–11.0)	2.5 (0.8–10.5)	0.894
Extremity cellulitis, n (%)	8 (4.7)	6 (4.8)	2 (4.8)	0.992
Any breast-related complication, n (%)	52 (30.6)	41 (32.3)	11 (25.6)	0.410
Any expander-related complication, n (%)	21 (12.4)	16 (12.6)	5 (11.6)	0.867
Any donor-site complication, n (%)	1 (0.6)	1 (0.8)	0 (0.0)	1.000
Unplanned reoperation, n (%)	41 (27.3)	32 (28.6)	9 (23.7)	0.559

**Table 4:** Multivariable logistic regression models for the impact of area deprivation index on outcomes

ADI	Lymphedema		Surgical complications		Reoperation	
	OR (95% CI)	*p*^a^	OR (95% CI)	*p*^b^	OR (95% CI)	*p*^c^
Q1-3	Reference		Reference		Reference	
Q4	5.10 (1.30–20.30)	**0.021**	0.67 (0.24–1.82)	0.428	0.80 (0.26–2.40)	0.676

^a^The model was adjusted for ethnicity, chronic obstructive pulmonary disease, and number of lymph nodes removed, ^b^The model was adjusted for ethnicity, diabetes mellitus, chronic obstructive pulmonary disease, preoperative chemotherapy, and radiation location, ^c^The model was adjusted for age, ethnicity, diabetes mellitus, hyperlipidemia, chronic obstructive pulmonary disease, preoperative and postoperative chemotherapy, and radiation location

### Postoperative therapy

While our protocol involves referral for both preoperative consultation/measurements and postoperative therapy/monitoring for all ILR patients, attendance of postoperative visits varied based on neighborhood deprivation. Patients in the highest ADI quartile (Q4) had a significantly higher rate of ‘no-shows’ compared to those in less deprived quartiles (Q1-3) (41.9% vs. 22.8%, *p* = 0.016).

### Patient-reported Outcomes

Of the 153 patients (118 in ADI Q1-3 and 35 in ADI Q4) with available email addresses who were sent questionnaires, 71 completed them with a 46.4% response rate (44.9% in ADI Q1-3 vs 51.4% in ADI Q4). Responders were distributed across ADI quartiles as follows: 31.0% in the lowest deprivation quartile (Q1), 23.9% in Q2, 19.7% in Q3, and 25.4% in the highest deprivation quartile (Q4). Patient-reported outcomes, assessed using the LYMPH-Q and stratified by ADI quartiles, are shown in Table 5. For all LYMPH-Q scales, a higher score represents a better outcome (e.g., fewer symptoms or better function). Patients residing in the most deprived neighborhoods (ADI Q4) reported lower median [IQR] scores across all assessed scales compared to those in less deprived neighborhoods (ADI Q1-3), specifically for symptoms (75.0 [63.9–87.5] vs. 88.9 [69.4–97.2]; *p* = 0.151), function (79.3 [65.5–98.3] vs. 96.6 [75.9–100.0]; *p* = 0.149), appearance (83.3 [60.0–100.0] vs. 100.0 [63.3–100.0]; *p* = 0.384), and psychological well-being (89.3 [64.3–100.0] vs. 96.4 [71.4–100.0]; *p* = 0.750), although these trends did not reach statistical significance. There was also no significant difference in the median [IQR] time from surgery to survey completion between the groups (41 [28.0–54.0] months for Q4 vs. 43 [29.0–54.0] months for Q1-3; *p* = 0.887).

**Table 5:** Patient-reported outcomes (LYMPH-Q) according to area deprivation index

Outcomes	Area deprivation index groups		*p* Value
	Q1-3	Q4	
	Median (interquartile range)		
Symptoms	88.9 (69.4–97.2)	75.0 (63.9–87.5)	0.151
Function	96.6 (75.9–100.0)	79.3 (65.5–98.3)	0.149
Appearance	100.0 (63.3–100.0)	83.3 (60.0–100.0)	0.384
Psychological	96.4 (71.4–100.0)	89.3 (64.3–100.0)	0.750
Time from surgery to survey completion, mo	43 (29.0–54.0)	41 (28.0–54.0)	0.887

A subgroup analysis of Black patients’ patient-reported outcomes according to the LYMPH-Q stratified by ADI quartiles was performed. Black patients residing in the most deprived neighborhoods demonstrated significantly lower median [IQR] scores in the function scale (62.0 [48.3–81.9] vs. 100.0 [100.0–100.0], *p* = 0.020), compared to those in less deprived neighborhoods. Similar trends were also seen across the symptoms (66.7 [46.5–83.3] vs. 94.4 [75.0–97.2]; *p* = 0.093) and psychological well-being (85.7 [54.5–100.0] vs. 100.0 [92.9–100.0]; p = 0.245).

## Discussion

This study demonstrated that patients residing in socioeconomically disadvantaged neighborhoods, as indicated by higher ADI scores, experienced a significantly increased risk of BCRL following ILR. After adjusting for competing risk factors, patients in the most deprived neighborhoods exhibited a fivefold higher risk of BCRL compared to those in less deprived neighborhoods. Notably, surgical complications and unplanned reoperations were not significantly affected by ADI status. Furthermore, while analysis of patient-reported outcomes across the entire cohort showed non-significant trends, a subgroup analysis revealed that neighborhood disadvantage significantly impacts functional quality-of-life among Black patients. These combined findings strongly suggest that neighborhood socioeconomic disadvantage attenuates the protective effect of ILR in reducing BCRL risk and is associated with worse patient-reported function in specific high-risk demographic groups, necessitating targeted interventions to mitigate disparities.

Our findings align with a growing body of literature highlighting the profound impact of socioeconomic factors, as measured by ADI, on patient outcomes [22, 23, 38–43]. ADI is a comprehensive measure that integrates multiple dimensions of socioeconomic disadvantage—such as income, employment status, educational level, insurance coverage, and housing quality [44, 45]. The mechanisms mediating the observed association between higher ADI scores and increased BCRL risk are likely multifaceted. Potential contributors include limited access to specialized healthcare providers experienced in lymphedema surveillance and management, potentially leading to delayed diagnosis and intervention. Furthermore, resource limitations often prevalent in disadvantaged areas may impede adherence to essential postoperative care regimens, such as consistent compression therapy and structured physical therapy, and hinder regular follow-up crucial for early detection. Indeed, further analysis showed a significant disparity in postoperative physical therapy attendance: 41.9% of patients residing in the most deprived neighborhoods (ADI Q4) did not attend their scheduled appointments (‘no-shows’), compared to 22.8% of patients in less deprived areas (ADI Q1-3) (*p* = 0.016). This difference provides direct evidence that inconsistent adherence to recommended therapy is more common among socioeconomically disadvantaged patients and likely contributes to the higher BCRL rates observed. This reliance on physical therapy visits for BCRL postoperative monitoring suggests that differential attendance likely also contributes to underdiagnosis of BCRL, particularly within high-ADI populations, potentially masking the full extent of the disparity. Collectively, these factors underscore the necessity of addressing structural barriers related to socioeconomic context to enhance the efficacy and equity of postoperative care pathways for breast cancer patients undergoing ILR.

Our study also identified a significant overrepresentation of Black patients within the highest ADI quartile. This demographic finding is particularly salient when considered alongside recently published findings demonstrating that Black patients experienced a significantly higher independent risk of developing BCRL following ILR compared to White patients [17]. The convergence of these findings—higher baseline clinical BCRL risk among Black patients, their concentration in high-ADI neighborhoods, and significantly worse patient-reported functional outcomes for Black patients in these high-ADI areas—points toward a critical intersectionality. Systemic disadvantages associated with deprived neighborhoods appear to exacerbate underlying racial disparities, impacting not only the clinical manifestation of lymphedema but also patients’ ability to perform daily activities. This complex interaction could reflect a combination of systemic inequities tied to geographic location, potential biological predispositions to lymphedema or impaired lymphatic healing in certain populations [46–50], and compounded barriers to accessing timely and consistent preventative care, including functional rehabilitation. Obesity and radiation are known risk factors for lymphedema. Mean BMI, obesity rate, and post-mastectomy radiotherapy were not significantly different between patients residing in high-ADI neighborhoods compared to other patients. Thus, these common risk factors would not account for the discrepancy of lymphedema rates. Understanding and addressing BCRL disparities therefore necessitates acknowledging this complex interplay between ethnicity and socioeconomic status.

While BCRL incidence was the primary focus, understanding the patient experience is also crucial. Although analysis of patient-reported outcomes using the LYMPH-Q across the entire cohort revealed non-significant trends toward worse outcomes in high-ADI neighborhoods, a focused subgroup analysis of Black patients demonstrated a statistically significant result. Specifically, Black patients residing in the most deprived neighborhoods demonstrated significantly lower scores on the LYMPH-Q function scale compared to Black patients in less deprived areas (62.0 vs. 100.0, *p* = 0.020). This scale assesses perceived difficulty with everyday tasks such as putting on clothes, washing hair, buttoning a shirt, reaching overhead, carrying groceries, and performing household chores [37]. These findings therefore imply tangible difficulties performing fundamental activities, highlighting a critical aspect of patient suffering directly linked to neighborhood disadvantage within this specific high-risk racial group. While trends toward worse symptoms and psychological well-being scores were also noted in this subgroup but did not reach significance, the pronounced functional impairment underscores the real-world impact of these disparities. This functional deficit may be driven by factors such as more severe or inadequately managed lymphedema, differential access to or uptake of physical and occupational therapy, or other unmeasured socioeconomic stressors prevalent in high-ADI areas that impact daily functioning. Validation of these subgroup findings and further investigation into the specific drivers of functional decline in this context are important avenues for future research.

While previous studies did not specifically examine the impact of socioeconomic factors on ILR outcomes, Chiang et al. [28]. provided compelling evidence for the underdiagnosis of BCRL in socioeconomically disadvantaged neighborhoods after breast cancer surgery. Using ADI, they found that individuals residing in more deprived areas had significantly lower odds of receiving a lymphedema diagnosis, despite having a higher prevalence of known risk factors for the condition. This strongly suggests that BCRL was underdiagnosed in socioeconomically disadvantaged populations prior to the widespread adoption of ILR. This context of probable underdiagnosis makes the significant difference in BCRL rates observed in our study between high- and low-ADI groups even more striking. It suggests that the true disparity in lymphedema burden associated with neighborhood disadvantage may be substantially greater than captured by current diagnostic practices. Furthermore, the significant functional deficits identified in our subgroup analysis emphasize that reliance solely on clinical diagnosis likely underestimates the true impact of lymphedema and associated disparities on patients’ lives. Prior studies established several mechanisms for the underdiagnosis of BCRL in socioeconomically disadvantaged populations, including lack of awareness and education about BCRL among these individuals. While the Lymphatic Education and Research Network has highlighted a general deficit in understanding lymphedema across all demographics, this is likely amplified in socioeconomically disadvantaged groups who may have lower health literacy or limited access to reliable health information [51]. We postulate that the lack of recognition of early symptoms, such as subtle swelling or heaviness in the affected limb, can lead to a failure to report these issues to healthcare providers, resulting in delayed intervention and potentially contributing to the higher BCRL rates observed in our study. Additionally, geographic barriers and transportation issues further compound these challenges. Socioeconomically disadvantaged individuals may reside in areas with limited availability of specialized medical centers capable of performing ILR or lack reliable transportation to attend numerous postoperative appointments with surgeons, oncologists, and lymphedema therapists, which could negatively impact the effectiveness of ILR and thus contribute to the higher rates of BCRL.

Financial toxicity associated with cancer care represents another significant barrier potentially amplified by neighborhood disadvantage [52, 53]. Variable insurance coverage for ILR in the United States and associated costs can impede access to the procedure and essential postoperative care, including physical therapy, compression garments, and follow-up appointments. While the Center for Medicare and Medicaid Services (CMS) has begun to cover some compression items [54], significant financial barriers and lack of access to qualified lymphedema therapists may still exist for many residing in disadvantaged neighborhoods. Inconsistent adherence to these critical components of care, driven by cost or access issues, could undermine the benefits of ILR and contribute to both higher incidence and greater functional impairment from BCRL. Consistent with this, Berlin et al. [55] found that women undergoing reconstructive procedures following mastectomy reported higher out-of-pocket medical expenses and a perceived decline in their financial status, a burden likely disproportionately affecting women in socioeconomically disadvantaged areas.

Addressing these disparities requires a coordinated, interdisciplinary approach involving policymakers, healthcare providers, community stakeholders, therapists, and patient advocacy groups. At the provider level, increasing awareness regarding the heightened risk of BCRL and potential for functional impairment among socioeconomically disadvantaged patients, particularly minority patients, is critical. Tailored educational programs that are culturally sensitive and multilingual, alongside the deployment of patient navigators trained in addressing socioeconomic and geographic barriers, could substantially enhance adherence to postoperative care recommendations, including timely referrals for functional therapy. Policy-level systemic reforms are essential to eliminate structural barriers to equitable access to ILR and postoperative care. Policies establishing standardized insurance guidelines ensuring comprehensive coverage of ILR procedures and associated follow-up care, including necessary rehabilitation services, may particularly help those underserved populations who disproportionately suffer from inadequate healthcare access. Community-driven interventions, such as telemedicine services, could further mitigate geographic and transportation barriers [56], ensuring timely access to specialized lymphedema care. Additionally, financial assistance programs facilitating affordable compression garments, transportation assistance, and accessible community-based lymphedema therapy services could significantly alleviate economic burdens on disadvantaged patients. Incorporating social workers and patient navigators into multidisciplinary care teams may further strengthen the linkage to essential community resources, promoting effective postoperative management without compromising patients’ financial stability. Specific attention to ensuring equitable access to and attendance at physical and occupational therapy is warranted given the functional deficits and attendance disparities identified.

This study has several limitations including the nonrandomized and retrospective design, which may introduce selection bias and confounding factors, potentially limiting the generalizability of our results. However, conducting the study across six hospitals involving diverse patient demographics enhances the robustness of our findings. The characteristics of our cohort, including a high prevalence of obesity, should be considered when generalizing our findings to populations with different baseline profiles. Furthermore, while we did not utilize diagnostic measures such as relative volume change, bioimpedance spectroscopy, or lymphography to define lymphedema, we employed two-point limb circumference measurements, previously reported as robust methods for identifying women at increased risk of developing permanent lymphedema.^33^ The sample size for the overall patient-reported outcome analysis was limited, and the subgroup analysis, while yielding significant findings for function, involved an even smaller number of patients, necessitating validation in larger studies. As LYMPH-Q data were collected postoperatively, we could not adjust for preoperative baseline functional status, which may have differed between groups. Additionally, this study did not capture data on patient-level factors such as postoperative physical activity levels or potential genetic predispositions, which have been suggested to influence lymphedema risk and represent unmeasured potential confounders. Future studies should address the limitations of the current study, ideally in the form of a prospective study that encompasses patient-reported outcomes in larger, diverse samples and evaluates the effectiveness of the proposed mitigation strategies in reducing BCRL incidence and functional disparities linked to neighborhood disadvantage after ILR.

## Conclusion

This study demonstrated a significant association between neighborhood socioeconomic disadvantage, measured by higher ADI scores, and an increased risk of BCRL following ILR, indicating a potential attenuation of ILR’s protective benefits in vulnerable populations. Importantly, subgroup analysis revealed that neighborhood disadvantage is also significantly associated with worse patient-reported functional quality-of-life among Black patients, highlighting a tangible impact on daily activities beyond clinical diagnosis. The relative concentration of Black patients, a group previously identified with higher baseline BCRL risk post-ILR, within high-ADI neighborhoods underscores the critical intersection of ethnicity and socioeconomic status in driving these disparities. These findings highlight the imperative for targeted, multifaceted interventions addressing neighborhood-level barriers (including therapy attendance and potential underdiagnosis), systemic inequities, access to functional rehabilitation, and potentially related biological factors to rectify existing disparities and optimize both clinical and patient-reported outcomes after lymphedema prevention surgery.

## Data Availability

No datasets were generated or analysed during the current study.
